# Methylation and Noncoding RNAs in Gastric Cancer: Everything Is Connected

**DOI:** 10.3390/ijms22115683

**Published:** 2021-05-26

**Authors:** Irina V. Bure, Marina V. Nemtsova

**Affiliations:** 1 Laboratory of Medical Genetics, Institute of Molecular Medicine, I.M. Sechenov First Moscow State Medical University (Sechenov University), 119991 Moscow, Russia; nemtsova_m_v@mail.ru; 2 Laboratory of Epigenetics, Research Centre for Medical Genetics, 115522 Moscow, Russia

**Keywords:** gastric cancer, noncoding RNAs, DNA methylation, epigenetics, biomarker

## Abstract

Despite recent progress, gastric cancer remains one of the most common cancers and has a high mortality rate worldwide. Aberrant DNA methylation pattern and deregulation of noncoding RNA expression appear in the early stages of gastric cancer. Numerous investigations have confirmed their significant role in gastric cancer tumorigenesis and their high potential as diagnostic and prognostic biomarkers. Currently, it is clear that these epigenetic regulators do not work alone but interact with each other, generating a complex network. The aim of our review was to summarize the current knowledge of this interaction in gastric cancer and estimate its clinical potential for the diagnosis, prognosis, and treatment of the disease.

## 1. Introduction

Gastric cancer (GC) remains the fifth most diagnosed cancer among both men and women and the third highest cause of cancer-related death worldwide. It affects large populations in the world, especially in East Asia, South America, and in the Far East; therefore, it can be considered as a socially significant disease [1,2].

Such a high mortality rate of GC is mostly explained by poorly pronounced and nonspecific symptoms at the early stages of the disease, leading to its diagnosis at advanced stages in around 85% of cases, at which point currently used methods of therapy became ineffective and the overall survival rate remains less than 25%. Furthermore, GC is heterogeneous disease with various clinical groups, characterized by different course, prognosis, and mechanism of development, which require subtype-specific treatment [3,4].

GC development is the result of a complex crosstalk among epigenetic, genetic, and environmental factors that lead to different molecular alterations in the cell [5,6]. Among environmental factors, the main role belongs to smoking, diet, and *Helicobacter pylori*, which cause a prolonged inflammatory reaction of the immune response [7,8]. Genomic DNA sequence alterations and mutations contribute to the deregulation of metabolic pathways and could lead to cancer initiation and progression [9]. Epigenetic changes affect oncogenes and tumor suppressor genes and, thus, lead to their over-expression or under-expression, respectively. By targeting apoptotic and DNA repair genes, as well as regulators of signaling pathways, transcription, and the cell cycle, they participate in the development and progression of GC [4]. Epigenetic mechanisms vary and include DNA methylation, histone modifications, chromatin remodeling, and alterations of noncoding RNA (ncRNA) expression. In contrast to environmental factors, which can be easily estimated and reduced, and genetic changes, which are stable, epigenetic modifications are both heritable and mostly reversible; however, they require an understanding of epigenetic regulation [10,11].

DNA methylation was one of the first discovered epigenetic alterations. To date, it has been characterized in numerous processes, both physiological and pathological, in all living things [12,13]. DNA methylation is a chemical modification, driven by the transfer of a methyl group from cofactor *S*-adenosylmethionine to the C5 position of the pyrimidine ring of a cytosine residue on DNA to form 5-methylcytosin [13]. It typically occurs in so-called CpG islands—regions with a high prevalence of CG dinucleotides located upstream of the most of mammalian promoters [14], and it is catalyzed by DNA methyltransferase enzymes (DNMTs). DNMT3A and DNMT3B are responsible for *de novo* DNA methylation, whereas DNMT1 maintains methylation during DNA replication [15]. Methylation of promoter regions determines tissue-specific gene expression, X-chromosome inactivation, and silencing of retroviral elements, and it alters the condensed chromatin structure by influencing histone–DNA or histone–histone contact [14]; deregulation of the methylation pattern can lead to serious diseases, including cancer.

Tumors are generally characterized by global DNA hypomethylation and hypermethylation of promoter CpG islands. Promoter hypermethylation may develop cancer by silencing tumor suppressor genes, involved in control of tumor-specific signaling pathways, DNA repair, cell cycle, and apoptosis, whereas DNA hypomethylation can contribute to genomic instability [16].

Conversely, ncRNAs represent a relatively “young” part of the epigenome that has only recently been considered as a comprehensive group of epigenetic regulators. The number of known ncRNAs in human is increasing, and, according to the Encyclopedia of DNA elements (ENCODE) reports, they constitute at least 76% of the genome, compared to less than 2% of protein-coding genes [17,18]. Their multiplicity, localization in transcriptionally active parts of the genome, and conservation across species further suggest their functional importance and make them valuable molecular candidates for diagnostic and therapeutic approaches for diseases, including cancer [19].

NcRNAs are represented by functional RNA molecules that are transcribed from DNA but not translated into proteins [4]. According to their location, length, structure, or biological functions, ncRNAs can be classified into different categories. There are housekeeping ncRNAs and regulatory ncRNAs. The first group includes ribosomal RNAs (rRNAs) and transfer RNAs (tRNAs) that were discovered earlier and participate in protein synthesis, while the second group includes ncRNAs localized in nucleus, namely small nuclear RNAs (snRNAs) and small nucleolar RNAs (snoRNAs) [20]. Regulatory ncRNAs are commonly classified on the basis of the length of their mature products into groups of long ncRNAs (>200 nt) and short ncRNAs (<200 nt), which are further classified on the basis of genomic origin and mechanism of action, including classes of microRNAs (miRNAs), short interfering RNAs (siRNAs), and PIWI-interacting RNAs (piRNAs) [4,21].

Currently, there are a number of studies devoted to the investigation of methylation pattern and ncRNA expression in all types of cancer. As part of the epigenome, these epigenetic regulators can not only regulate the expression of genes, but also mutually influence each other via different mechanisms, forming a complex regulatory network. An understanding of these mechanisms and correlations could significantly enrich our knowledge about tumorigenesis and have practical importance. Abnormal DNA methylation patterns and deregulated ncRNAs are more common than genetic alterations and occur very early during carcinogenesis; they are tumor-specific and significantly correlate with cancer progression and pathological alterations in patients, making them prominent diagnostic and prognostic biomarkers. Additionally, as part of epigenome, they are normally reversible and, hence, could also be potential targets for therapeutic agents [22].

The aim of this review was to bring together the current knowledge and research on the interplay between DNA methylation and ncRNAs in GC and their clinical potential for the diagnosis, prognosis, and treatment of the disease.

## 2. Methylation and miRNAs: Feedback Loops

MicroRNAs (miRNAs) are a class of evolutionarily conserved small noncoding transcripts of 20–24 nucleotides in length that regulate expression of their target genes at the post-transcriptional level by either repressing translation or causing messenger RNA (mRNA) degradation [23].

Discovered in *C. elegans* in 1993 [24], they are the first described and most comprehensively investigated group of regulatory ncRNAs. Currently, 2654 mature human miRNAs have been described (miRbase, release 22.1, October 2018) [25]; they are expressed in virtually all tissues at all stages of development, and they are able to target multiple transcripts in all types of cells [26].

Biosynthesis of miRNAs is a multistep process that proceeds in both the nucleus and the cytoplasm and involves several proteins and enzymes. RNA polymerase II synthesizes a precursor that is further converted into a primary transcript (pri-miRNA). The RNase III endonuclease Drosha processes it into a precursor hairpin miRNA (pre-miRNA), which is subsequently transported to the cytoplasm by exportin and further processed by another endonuclease Dicer. The resulting miRNA duplex is unwound by helicases, and a mature miRNA is incorporated into the RNA-induced silencing complex (RISC) that directs it to the target mRNA [22,26].

MiRNAs negatively regulate the expression of specific genes through binding to the mRNA 3’ untranslated region (UTR) in their recognition sites at nucleotides positioned 2–7 from the 5′, called the “seed sequence” [27]. Depending on the degree of complementarity, this results in either translational repression (partially complementary) or mRNA cleavage (perfectly complementary) [22]. In some cases, the 3’ end nucleotides 13–16 of the miRNA can also be involved in target recognition as a “supplemental region” [28]. MiRNAs are involved in processes of development and differentiation, cell-cycle progression, proliferation, and apoptosis, and their deregulation is one of the key factors in the origin and progression of many tumors, including GC [29,30].

A number of studies have demonstrated that miRNAs are related to GC development, progression, and response to treatment, acting as oncogenic or tumor-suppressive regulators [22,29]. For example, the miR-200c is downregulated in GC and represses E-cadherin through targeting *ZEB1*, which leads to poorly differentiated histology in GC cells [31]. E-cadherin is an important player in the epithelial–mesenchymal transition (EMT) process, which is the main mechanism that determines invasion and metastasis of cancer cells. Therefore, miRNAs that directly or indirectly suppress *CDH1* assist EMT [32].

MiRNAs are the one of the largest classes of epigenetic regulators. However, they can also be regulated by epigenetic mechanisms, including DNA methylation, with dysregulation of miRNA methylation being a hallmark of cancer initiation and metastasis [22]. Recent studies have shown that about 50% of miRNA genes are associated with CpG islands; thus, their expression in a cancer-specific manner can be influenced by DNA methylation [33]. Previous studies have demonstrated that, similarly to the protein-coding genes, tumor-suppressive miRNAs can be silenced with abnormal hypermethylation at their promoter regions, which is commonly associated with a malignant phenotype [34]. Thus, the expression of miRNA-34b and miRNA-129 is frequently reduced in GC cell lines with CpG-rich methylation, and their downregulation was significantly modulated by upstream CpG island hypermethylation and correlated with poor clinical outcome in GC patients [35]. Tumor-suppressive miR-9 regulates cell proliferation, migration, and invasion in normal condition and is significantly downregulated in GC in comparison with adjacent normal tissues. Tendency toward a specific DNA methylation pattern was demonstrated for all three independent genetic loci miR-9-1, miR-9-2, and miR-9-3 in primary human gastric cancer specimens [36,37,38]. A number of studies have demonstrated that the miRNA miR-125a-5p is an important tumor suppressor in many tumors and is one of the most downregulated miRNAs in GC tissues [39,40,41,42]. In normal tissue, it targets oncogenes vascular endothelial growth factor A21 and E2F transcription factor 3.22, thus inhibiting cell proliferation and migration and preventing GC development [39,40]. However, miR-125a-5p was only recently found to be embedded in CpG islands and hypermethylated in GC tissues [39], similarly to previously studied glioma cells [43]. This means that miR-125-5p expression is regulated via epigenetic mechanisms, including not only DNA methylation, but also histone deacetylases (*HDACs*) and methyltransferase *Suv39H1* [39]. Furthermore, Cai et al. demonstrated that histone methyltransferase *Suv39H1* is the target gene of miR-125a-5p, which means that over-expressing miR-125a-5p could self-activate the silenced miR-125a-5p in GC cells, resulting in cancer suppression in vitro and in vivo [39].

Regulation by DNA methylation was also confirmed for another tumor-suppressive miRNA miR-31, whose expression was significantly decreased in GC tissue and cell lines. Ectopic expression of miR-31 potentially suppresses proliferation and induces early apoptosis in GC cells. Additionally, it is involved in an epigenetic feedback loop through directly targeting oncogenic *HDAC2* [44]. miRNA CpG hypermethylation was also found in GC mucosae with *H. pylori* infection, showing a significantly higher level than that in normal gastric mucosae [45].

MiRNAs also contribute to epigenetic regulation, when they directly target and, thus, post-transcriptionally suppress the mRNAs of genes involved in the DNA methylation process, such as DNA methyltransferases [22]. Promoters of miRNA target genes are often negatively correlated with DNA hypomethylation, and DNA methylation is regulated by DNMTs. It was demonstrated that *DNMT3a* and *DNMT3b* are commonly over-expressed in tumors with poor prognosis, and they are directly or indirectly targeted by a subset of miRNAs in different types of cancer [22,46]. Thus *DNMT3a* is also a direct target of miR-143 [47], while members of the miR-29 family target *DNMT3a* and *DNMT3b* [48] and can also indirectly repress *DNMT1* [49]. *DNMT1* was confirmed as a target of miR-152 and miR-148a [50]. Moreover, miR-148a was identified as a regulator of a DNMT3b splice variant through binding to the coding region in GC [51].

Sometimes, the regulation of miRNAs by methylation and vice versa can occur in the same miRNA, appearing as a feedback mechanism. This type of regulation was first described for miRNA-212 by Wada et al. Their investigation revealed that miRNA-212 is downregulated in both GC tissue and cell lines, targets the *MECP2* gene, and represses the construct with the MECP2 3’ UTR. Over-expression of miRNA-212 downregulated an important component of DNA methylation (MECP2 protein expression), resulting in lower DNA methylation, and inhibited GC cell proliferation. It was also further found that miRNA-212 influenced DNA methylation by regulating MECP2 at the translational level [52].

**Figure 1:**
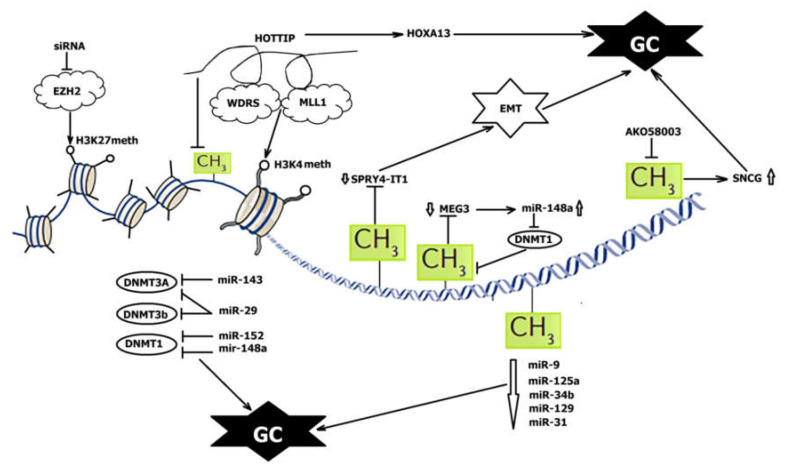
The interplay between DNA methylation and ncRNAs in GC epigenetic regulation. GC—gastric cancer; EMT—epithelial–mesenchymal transition; arrows—activation; bars—suppression.

Some mechanisms of miRNA and DNA methylation interactions are presented in Figure 1.

Being able to inhibit genes involved in DNA methylation processes and being repressed by DNA methylation, miRNAs are involved in regulatory feedback loops. This complex epigenetic mechanism significantly influences gene expression level, and its deregulation can destroy normal physiological processes and lead to tumorigenesis [22].

## 3. DNA Methylation and lncRNAs: Extending the lncRNA–miRNA–mRNA Pathway

Long noncoding RNAs (lncRNAs) include transcripts longer than 200 nt with no or limited protein-coding capacity [9], and they have many characteristics, similar to mRNAs. Thus, they are transcribed by RNA polymerases I, II, or III, capped, spliced, and polyadenylated [53], and can have a 3’ UTR region and K4–K36 domain of active promoters (H3K4me2/3, H3K9ac, H3K27ac), as well as actively transcribed gene bodies (H3K36me3) [4]. However, there are also several important differences, including shorter length with a smaller number of exons, lower expression level, much higher tissue-specificity, and absence of an open reading frame (ORF) [54]. The NONCODE database estimates 96,411 lncRNA genes and 173,112 lncRNA transcripts in the human genome [55].

LncRNAs can be classified into nonoverlapping and overlapping lncRNA. The first group includes intergenic lncRNAs (lincRNAs) that are located at least 5000 nt away from protein-coding genes and transcribed completely from intergenic regions [56]. Overlapping lncRNAs include intronic lncRNAs (iRNAs) that are transcribed from introns of protein-coding genes and antisense lncRNAs (asRNAs), related to the RNA sequence complementary to protein-coding mRNA [57].

Currently, it is clear that lncRNAs play an important role in a variety of biological processes, including cellular differentiation, integrity of cellular structures, cell-cycle regulation, intracellular trafficking, chromatin imprinting, and chromosomal dosage compensation [4]. Being localized in the nucleus and cytoplasm, lncRNAs regulate genes both transcriptionally and post-transcriptionally [58]. Their structural heterogeneity and complex secondary and tertiary structures enable them to act via different mechanisms and perform variable functions in the cell [54,59].

LncRNAs can act as guides to direct the localization of the ribonucleoprotein, resulting in the expression of genes loading in *cis* or in *trans*, as decoys, by binding to regulatory proteins and preventing their interaction with regulatory elements, or as scaffolds, serving as intermediates in the connection of two or more proteins into one complex [4,60]. LncRNAs can also interact with mRNAs, regulating their translation or acting as miRNA sponges [57]. Moreover, lncRNAs are important players in epigenetic regulation involved in histone modification, DNA methylation, genomic imprinting, and chromatin remodeling [61]. Deregulation of lncRNAs can lead to blockage of signaling pathways, and it has already been described in numerous pathological states including GC [62].

The best studied lncRNA HOTAIR, described as oncogenic in many types of cancer, is also over-expressed in GC, leading to the promotion of cell growth and invasion [63,64]. Together with PRC2, it could inhibit miR-34a and induce EMT [65]. HOTAIR may also promote metastasis via PCBP1 [66], and it is correlated with shorter overall survival of GC patients [64]. LncRNA expression in GC could also interact with *H. pylori* infection, as the high-salt diet of these patients promotes lnc-SGK1, thus affecting the Jun-B signaling pathway and triggering Th2 and Th17 cell differentiation [67]. *H. pylori* infection also inhibits tumor suppression lncRNA AF147447 via E2F1-dependent silencing of its transcription promoter, thus also adding to GC progression [68].

As protein-coding genes, genes of lncRNAs have promoter regions and can be regulated by DNA methylation in a similar manner [69]. To date, few DNA methylation-regulated lncRNAs have been investigated in GC, such as LOC100130476, GAS5, SPRY4-IT1, and MEG3. Their hypermethylation in GC was confirmed by their reactivation upon treatment of GC cells with DNA methylation inhibitors [4].

LOC100130476 is downregulated in GC tissues and cells and is associated with pathological differentiation, TNM stage, and survival in GC patients. The methylation of LOC100130476 was described in both exons; however, the methylation pattern of region 1 nearest to the transcription start site is more tumor-specific. The aberrant methylation of CpG sites within exon 1 is critical for LOC100130476 expression and causes poor prognosis [70].

LncRNA SPRY4-IT1 is downregulated in GC, and its expression is associated with tumor size, advanced pathological stage, depth of invasion, lymphatic metastasis, and poor prognosis. SPRY4-IT1 is transcribed from the second intron of the *SPRY4* gene and involved in the regulation of EMT. It was found that the promoter region of the SPRY4-IT1 loci has CpG islands. Treatment of GC cells with DNA methylation inhibitor 5-aza-CdR significantly upregulated SPRY4-IT1 expression, and the same effect led to the knockdown of *DNMT1*. Therefore, SPRY4-IT1 expression is regulated by DNMT1-mediated DNA methylation [71]. Furthermore, silencing of SPRY4-IT1 suppresses the progression of GC by sponging miR-101-3p and, thus, regulating *AMPK* expression [72]. The promoter region and the intergenic germ line-derived differentially methylated region (IG-DMR) of lncRNA MEG3 are rich in CpG dinucleotides, and their DNA methylation plays an important role in MEG3 downregulation in GC. The ectopic expression of MEG3 could inhibit cell proliferation, promote cell apoptosis, and modulate p53 expression in GC cell lines. The low expression of MEG3 is significantly correlated with invasion and tumor size [73]. It is also positively correlated with miR-148a expression levels through regulating *DNMT1* expression. miR-148a suppresses tumorigenesis by inhibiting DNMT1-dependent DNA methylation in GC. It was suggested that miR-148a suppression also contributes to MEG3 downregulation via DNMT1 activation [4] (Figure 1). Promoter methylation also plays an important role in the decreased expression of lncRNAs GAS5 [74,75,76].

LncRNAs are known as a major epigenetic modifier of chromatin states [4]. However, they can also mediate epigenetic regulations in GC through DNA methylation regulation. The already mentioned lncRNA HOTAIR promotes CpG methylation in the promoter region of the tumor suppressor gene *PTEN*, resulting in its downregulation and, thus, promoting EMT in GC [77].

Two intronic antisense lncRNAs AK058003 and AK123072 were described to be upregulated under hypoxic conditions and enhance GC cell migration and invasion by decreasing CpG island methylation in metastasis-associated genes, thus upregulating their expression [78,79]. Additionally, AK123072 increased GC metastasis [76]. It was experimentally confirmed that AK058003 and AK123072 knockdown significantly increased the methylation of CpG islands in their proposed targets *SNCG* and *EGFR*, respectively [78,79].

LncRNA HOTTIP (HOXA transcript at the distal tip) is over-expressed in GC cells. It is located at the 5’ end of the *HOXA* locus and regulates activation of several *HOXA* genes by recruiting PRC2 and the WDR5–MLL complex to the 5’ end of the *HOXA* cluster, resulting in H3K4 methylation and transcriptional activation of the *HOXA* locus, including the *HOXA13* gene, whose increased expression contributes to tumorigenesis. It was demonstrated that the recruitment of MLL1 and WDR5 and the trimethylation of histone H3 lysine 4 (H3K4me3) occur at the CpG position of the *HOXA13* promoter in GC cell line CS12. Moreover, reduced DNA methylation at this site is also observed with the restriction of DNMT1 and DNMT3b recruitment to the E1 site. HOTTIP suppression restores the recruitment of DNMT3b, but not DNMT1 [4,80] (Figure 1).

The antisense lncRNA HOXA11-AS is highly expressed in GC, where it regulates cell growth, migration, invasion, and apoptosis, and it is associated with poor prognosis. Several studies described different presumable ways of HOXA11-AS regulation. Thus, it can interact with genes such as *CDH1*, *E2F1*, and *CDKN1A* (*P21*) [81] or recruit EZH2 along with DNMT1, mediating DNA methylation [4]. The antisense lncRNA PYCARD-AS1 can also recruit DNMT1 and histone methyltransferase G9a to the PYCARD promoter to regulate apoptosis [82].

LncRNA MLK7-AS1 is over-expressed in GC and correlates with shorter survival and poor prognosis. It was demonstrated that knockdown of MLK7-AS1 inhibits cell proliferation and induces apoptosis in GC cells. Furthermore, miR-375 was identified as a target of MLK7-AS1. MLK7-AS1 interacted with DNMT1 and recruited it to the miR-375 promotor, thus resulting in its hypermethylation and repression of miR-375 [83].

Thus, lncRNAs can both be regulated by DNA methylation and regulate DNA methylation. In this process, some lncRNAs interact with miRNAs and the lncRNA–miRNA–mRNA pathway then includes one more step of epigenetic regulation before modifying target genes in GC tissues [4].

## 4. DNA Methylation and siRNAs: Retrotransposon Silencing

Small interfering RNAs (siRNAs) are transcripts around 21–22 nt that are involved in gene regulation, control of transposons, and viral defense [84]. As in miRNAs, their biogenesis includes cleavage by endonuclease Dicer; however, they are derived from long double-stranded RNA molecules, including RNAs arising from virus replication, transposon activity, or gene transcription. Double-stranded siRNA duplexes are further separated by helicase, and mature double-stranded siRNAs operate in an RISC complex, where Argonaute proteins have a catalytic function. SiRNAs form a perfect duplex with their mRNA targets and direct the RISC-mediated cleavage at the site of complementarity [85].

It was recently demonstrated that siRNAs can influence DNA methylation and histone modification with consequent transcriptional gene silencing [21,86]. By silencing histone methyltransferase EZH2, siRNA could prevent H3K27 methylation and consequent CpG methylation, thus regulating gene expression and reversing cisplatin resistance in GC cells [87] (Figure 1).

Several teams have demonstrated that depletion of Dicer and the key components of RISC Argonaute and Rdp1 can cause the aberrant accumulation of lncRNAs, resulting in the loss of H3K9me, thus impairing centromere function [21]. Additionally, the development of sequencing technology revealed a correlation between H3K9 methylation and repetitive elements, which occupy two-thirds of the human genome. This suggests the possibility that RNA interference pathways may have a more impressive role in regulation of the epigenome [21]. SiRNAs can also induce de novo DNA methylation and add a methyl groups to homologous DNA sequences, being incorporated in small-RNA-guided epigenomic editing complexes. This process, known as RNA-directed DNA methylation (RdDM), was described in plant cells and found in human cells; however, it has not been evaluated clearly [88].

The epigenetic regulation of long interspersed nuclear element 1 (LINE-1) retrotransposons by siRNAs was also described in cancer. In contrast to normal cells, where the LINE-1 retrotransposons are mostly repressed, cancer cells are characterized by their aberrant expression, which could contribute to genomic instability. Using deep-sequencing small-RNA analysis, Chen et al. identified a subset of differentially expressed siRNAs that directly regulate LINE-1 expression. The over-expression of these siRNAs in breast cancer cells significantly silenced endogenous LINE-1 expression through increased DNA methylation of the LINE-1 5′ UTR promoter [89].

Therefore, it seems that further investigation of siRNAs may lead to a deeper understanding of epigenetic regulation.

## 5. DNA Methylation and piRNAs: Novel Candidate?

Piwi-interacting RNAs (PiRNAs) are another class of regulatory small RNAs with noncoding transcripts of 25–30 nt, and they are considered as the most numerous class of ncRNAs [90].

In contrast to miRNAs, piRNAs are generated from single-stranded precursors (pre-piRNAs) via a Dicer-independent maturation process and transcribed bidirectionally [27]. PiRNA clusters can be inserted into pseudogenes, as well as intergenic and protein coding-regions [91], and they are proposed to be the most abundant class of ncRNAs, with more than 30,000 piRNAs described in the human genome [92].

The functions of piRNAs still remain mostly unknown. The main functions of piRNAs are thought to be the silencing of transposable elements and the regulation of chromatin state in germline cells by binding the PIWI subfamily of Argonaute proteins [91]. However, the presence of piRNAs in protein-coding regions suggests that they could also be involved in the regulation of protein-coding mRNAs [93]. PiRNAs demonstrated regulatory function at both the transcriptional and the post-transcriptional level [94].

In 2011, piR-015520 was the first human piRNA found regulating a protein-coding gene, and it was associated with the regulation of the *MTNR1A* gene [95]. Further investigation confirmed that piR-015520 is also involved in the epigenetic regulation of genes of embryonic and gonadal development, sex determination, gametogenesis, apoptosis, and stem-cell division [91].

PiRNAs can also regulate gene expression through post-transcriptional inhibition of mRNA translation by incomplete base pairing. As piRNAs act as regulators of the genome, their aberrations may have a role in tumorigenesis. Potential interactions between piRNAs and other noncoding RNAs have also been suggested [91].

An investigation of the piRNA expression profile in GC revealed piR-651 over-expression in tumor tissue and cell cultures. The positive correlation of piR-651 expression with advanced stages in GC patients, as well as proliferation in MGC-803 and SGC-7901 cell lines, was shown [96]. Moreover, piR-651 was found in the peripheral blood of GC patients, making it a potential biomarker of GC. Conversely, piR-823 was found to be downregulated in GC cells, and normalization of the piRNA level decreased GC cell proliferation [91,94].

PiRNAs in complex with PIWI proteins and epigenetic factors cause histone modifications, acting as a guide [94]. They have also been shown to directly regulate DNA methylation, making them a functional element of epigenetic regulation in cells [97]. Piwi proteins MILI and MIWI2 were found to play an important role in methylation and silencing of transposons LINE-1 and IAP in the testis by regulating DNMT3a and DNMT3b [98]. The piRNA-mediated regulation of DNA methylation in GC remains poorly understood; however, piRNAs are considered prominent candidates for further investigation [94].

The interaction of ncRNAs of different classes with DNA methylation, described in this review, are summarized in the Table 1.

## 6. Methylation and ncRNAs in GC Diagnostics and Treatment

Despite the progress in diagnosis and treatment, GC remains a tumor with poor prognosis and survival because of its predominant diagnosis at an advanced stage among patients. The diagnosis of GC is largely based on gastroscopic screening, which is reliable, but invasive, whereas commonly used blood serological markers carbohydrate antigen 19-9 (CA19-9) and carcinoembryonic antigen (CEA) demonstrate a lack of sensitivity and specificity [106]. Therefore, biomarkers for early detection and therapeutic targets for effective treatment are critically needed, and epigenetic regulators could be prominent candidates.

The detection of promoter hypermethylation as an epigenetic marker may be an efficient method for tumor diagnostics. For example, the promoter of *p16* (*INK4a*) is hypermethylated in GC; therefore, *p16* methylation could be a candidate biomarker for detection of gastric cancer [107]. Methylated ncRNAs of different classes are also applicable to GC diagnostics [62].

MiRNAs as biomarkers have a number of advantages. They are stable in body fluids and are easily obtained, making them suitable for noninvasive diagnostics [108]. They are also stable in high-temperature, long-term storage, and strong acidic or basic conditions; therefore, they can be detected in a wide range of samples, including formalin-fixed paraffin-embedded (FFPE) samples [109]. It was also proposed that miRNA methylation could potentially be a more accurate cancer diagnostic tool than miRNA expression [33]. However, up to now, only few miRNAs have been approved for clinical use and none of them in gastric cancer. Among the potential biomarkers are miR-22, associated with GC development [110], miR-125a-3p, whose low expression correlates with tumor size, metastasis, invasion, and poor prognosis [106], and a panel of three miRNAs miR-221, miR-20a, and miR-106b, whose level in plasma was significantly increased in GC patients in comparison to normal controls [111]. MiRNA expression could also be associated with drug resistance in GC. Thus miR-132 was demonstrated to promote cisplatin resistance in GC cells through SIRT1/CREB/ABCG2 signaling pathway regulation [112], and miR-106a contributed to the decreased sensitivity of GC cells to anticancer drugs and restrained drug-induced apoptosis by inducing the expression of antiapoptotic *BCL-2* [113]. Therefore, over-expression of these miRNAs could be a prognostic biomarker for drug resistance.

The possible application of epigenetic regulators in GC treatment has also been considered. One of the prominent methods involves DNA demethylation therapy. Aberrant DNA methylation of promoter CpG islands commonly leads to silencing of tumor-suppressing transcripts [114]. However, this process could be reversed by DNA methyltransferase inhibitors (DNMTis), including FDA-approved 5-azacytidine (azacitidine) and 5-aza-2-deoxycytidine (decitabine) that inhibit DNA methyltransferases, thereby inducing replication-dependent DNA demethylation following re-expression of genes [115,116]. It was also demonstrated that pretreatment with azacitidine increased the efficacy of neoadjuvant chemotherapy with EOX (epirubicin, oxaliplatin, capecitabine) in patients with resectable gastric adenocarcinoma [117].

Solid tumors demonstrate variable responses to DNA-demethylating agents [118]. Therefore, identification of patients able to respond and gain benefit from DNA demethylation therapy is important. Thus, in GC cells, an association between sensitivity to azacitidine and CpG island methylator phenotype (CIMP) has been reported [115]. For example, lncRNA LINC00162 may have translational value to predict patients who will respond to azacitidine. It is highly and frequently expressed in GC cell lines sensitive to 5-aza-2′-deoxycytidine, and its over-expression increases sensitivity. LINC00162 enhances cell-cycle arrest and apoptosis induced by azacitidine through affecting antiapoptotic splicing variant BCL-XL, but it does not affect the DNA demethylation effect [115]. There are already a number of lncRNAs described as regulators of GC resistance to drugs, including doxorubicin, cisplatin, and fluorouracil [119]. Among them are HOTAIR, which can activate the PI3K/AKT/MRP1 pathway and, thus, increase the resistance of GC cells to cisplatin [120], and ANRIL, which determines resistance to cisplatin and 5-fluorouracil [121]. Therefore, they seem to be prominent therapeutic targets.

One of the commonly used strategies to decrease lncRNA level in tumor cells is knockdown by siRNAs or short hairpin RNA (shRNA). Both of them silence lncRNAs via RNA interference, are transported to the cells by transfection with plasmid vectors, and have off-target effects [122]. Antisense oligonucleotides (ASOs) are also used to affect lncRNAs. They are single-stranded, and they have native or a phosphorothioated DNA stretch at the central part and RNA nucleotides at the ends. Upon interacting with target lncRNA, they form an RNA/DNA heteroduplex, which is further cleaved by endogenous RNaseH1 [123]. Furthermore, similar regulatory structures were elaborated by modifications, e.g., locked nucleic acid GapmeRs (LNA GapmeRs) with chemically modified LNA in flanking regions to increase binding affinity [124], antagonist to NATs (antagoNAT), for targeting natural antisense lncRNAs (NATs) [125], and mixmers that could not be degraded by RNase H1 and were used to sterically inhibit the linkage, connecting the lncRNAs with ribonucleoproteins or nucleic acids [126].

Modification of miRNA expression in GC cells may also be a potential treatment strategy against tumors. Transfection experiments in vitro were conducted in many studies and demonstrated a clear effect. For example, ectopic expression of miR-141 in tumor cells may lead to a ~40% inhibition of proliferation and significant reduction in invasion [127]. Experiments in vivo are much rarer, but they also demonstrated prominent results. Thus, the injection of miR-29c mimicked intratumorally inhibited tumor growth and exerted antimetastatic effects in a xenograft nude mouse model [128].

The strategy of therapeutic miRNA manipulation in cancer depends on its function. The oncogenic miRNAs that are over-expressed in tumors need to be suppressed, whereas tumor-suppressive miRNAs require restoration of their expression [129]. As some miRNAs directly target DNMTs, affecting them also means affecting DNA methylation [130].

The most common therapeutic approach is the application of chemically modified oligonucleotides, i.e., anti-miRNA oligonucleotides (AMOS), that perfectly match to the mature target miRNA, thereby titrating away the functional transcript or synthetic double-stranded miRNA mimics that are complementary to the mRNA target and useful to restore the expression of tumor-suppressor miRNAs [131]. Several such oligonucleotides have been already included in clinical trials as candidate therapeutics in different types of cancer [132,133]. Another approach to silencing oncogenic miRNAs considers sponges transcribed from mammalian expression vectors that contain multiple binding sites to an miRNA of interest [134]. Because of this feature, sponges allow binding an entire miRNA family [22] or even several different miRNAs simultaneously [135], collecting them into a nonfunctional complex and, thus, sequestering the mature free miRNA target. Another option to reduce the expression of oncogenic miRNAs is the use of miR-masks, which bind to the 3’ UTR sites of the target mRNAs and block targeting by miRNAs, thus interacting not with the miRNA, but with the gene [22].

Different classes of ncRNAs have demonstrated their potential for structural modification, which may be beneficial in therapy [62]; however, their application in practice still has serious restrictions. Thus, miRNA expression is variable in different conditions, which can result in biases even when a panel of several transcripts is used [22]. LncRNA profiling demonstrates significant regional and rational differences; thus, it seems that no universal diagnostic and prognostic biomarkers will be found [62]. The application of ncRNAs in therapy is even more challenging. Thus, the advantage of miRNA-based therapeutics is the fact that one single miRNA can affect multiple targets; however, this may also lead to serious side-effects [129]. Their delivery systems also require further optimization [22,106]. Three therapeutic approaches are currently in development, namely, lipid components, viruses, and nanoparticles [106,136]. Nevertheless, most studies suggest the clinical application of epigenetic regulators upon thorough investigation.

## 7. Conclusions

Gastric cancer is a widespread and aggressive disease with poor prognosis. Epigenetic regulators, such as DNA methylation and ncRNAs, play a significant role in disease development and prognosis; however, they may also become novel prominent biomarkers for early diagnosis and effective therapeutic targets. In the last few years, it has become even clearer that epigenetic regulators are tightly connected and form a comprehensive network of regulatory pathways and feedback loops. This is particularly interesting for a better understanding of processes that occur in GC tumorigenesis, as well as approaches for their possible reparation. Therefore, in this review, we summarized data about the roles of different epigenetic regulators involved in GC by focusing on DNA methylation and its mutual regulation with ncRNAs. Their potential application in GC diagnosis and treatment was also described. Numerous investigations have been conducted leading to many findings in the field of epigenetic regulation. Nevertheless, the practical application of this knowledge remains a challenge, but it is likely to advance in the future.

## Figures and Tables

**Table 1:** Mutual epigenetic regulation of ncRNAs and DNA methylation in GC.

NcRNA	Status in GC	Interaction with DNA Methylation, Function in GC	References
**DNA methylation regulates ncRNAs**			
miRNA-34b miRNA-129-3p	Down	Methylated; associated with poor prognosis	[35]
miR-9	Down	Methylated; regulates cell proliferation, migration, and invasion	[36,37,38]
miR-125a-5p	Down	Methylated; directly targets *HDACs*; regulates cell proliferation and migration	[39,40]
miR-31	Down	Methylated; involved in epigenetic feedback loop through directly targeting oncogenic *HDAC2*; regulates proliferation and apoptosis	[44]
miR-124-1 miR-124-2 miR-124-3	Down	Hypermethylated in GC mucosae with *H. pylori* infection	[45]
miR-512-5p	Down	Methylated; activated upon DNA demethylation at Alu repeats; suppresses *Mcl-1*, resulting in apoptosis	[99]
miR-181c	Down	Methylated; targets oncogenes *NOTCH4* and *KRAS*	[100]
miR-129-2	Down	Methylated; targets *SOX4* and, thus, regulates apoptosis	[101]
miR-137	Down	Methylated; targets *Cdc42* and, upon reactivation, induces apoptosis and cell-cycle G1 arrest in gastric cancer cells	[102]
miR-139	Down	Methylated; regulates metastases through *HER2*, *CD44*, and *CXCR4*	[103]
miR-148a	Down	Methylated; targets *DNMT1*	[51]
miR-155	Down	Methylated; involved in cell metastasis	[104]
miR-195	Down	Methylated; suppresses *CDK6* and VEGF signaling	[105]
miR-378a			
miRNA-212	Down	Methylated; targets *MYC* and, thus, participates in tumorigenesis	[52]
SPRY4-IT1	Down	Regulated by DNMT1-mediated DNA methylation; regulates proliferation, invasion, and EMT	[71]
MEG3	Down	Promoter methylation; regulates proliferation and apoptosis; modulates p53 expression; correlates with invasion and tumor size	[73]
GAS5	Down	Promoter methylation; regulates proliferation and adriamycin sensitivity; correlates with poor prognosis	[74,75]
HOXA11-AS	Up	DNA methylation; regulates proliferation and invasion by scaffolding PRC2, LSD1, and DNMT1	[81]
LOC100130476	Down	Methylation in the CpG islands; associated with pathological differentiation, TNM stage, and survival	[70]
**NcRNAs regulate DNA methylation**			
miR-148a	Down	Regulator of a *DNMT3b* splice variant; directly targets *DNMT1*	[50,51]
miR-143		Directly targets *DNMT3a*	[47]
miR-29		Directly targets *DNMT3a* and *DNMT3b*; indirectly targets *DNMT1*	[48,49]
miR-152		Directly targets *DNMT1*	[50]
HOTAIR	Up	H3K27 trimethylation; inhibits miR-34a and induces EMT; promotes metastasis; correlated with shorter survival	[64,65,66]
HOTTIP	Up	Methylation in the CpG islands H3K4 methylation HoxA13 suppression restores the recruitment of DNMT3b	[4,80]
AK058003	Up	Regulates methylation of CpG islands in *SNCG*; promotes migration, invasion, and metastasis	[79]
AK123072	Up	Regulates methylation of CpG islands in *EGFR*; promotes migration and invasion	[78]
PYCARD-AS1	Down	Recruit DNMT1 and histone methyltransferase G9a to the *PYCARD* promoter to regulate apoptosis	[82]
MLK7-AS1	Up	Regulates proliferation and apoptosis; interacts with DNMT1 and recruits it to miR-375, resulting in its hypermethylation and repression of miR-375; correlates with poorer prognosis	[83]

Up—upregulated; down—downregulated.
